# The Glare Effect Test and the Impact of Age on Luminosity Thresholds

**DOI:** 10.3389/fpsyg.2017.01132

**Published:** 2017-06-30

**Authors:** Alessio Facchin, Roberta Daini, Daniele Zavagno

**Affiliations:** ^1^Department of Psychology, University of Milano-BicoccaMilan, Italy; ^2^Milan Center for NeuroscienceMilan, Italy; ^3^Optics and Optometry Research CentreMilan, Italy

**Keywords:** glare effect, illusion sensitivity, aging, perception

## Abstract

The glare effect (GE) is an illusion in which a white region appears self-luminous when surrounded by linearly decreasing luminance ramps. It has been shown that the magnitude of the luminosity effect can be modulated by manipulating the luminance range of the gradients. In the present study we tested the thresholds for the GE on two groups of adults: young (20–30 years old) and elderly (60–75 years old). Purpose of our perspective study was to test the possibility of transforming the GE into a test that could easily measure thresholds for luminosity and discomfort glare. The Glare Effect Test (GET) consisted in 101 printed cards that differed from each other for the range of luminance ramps. Participants were assessed with GET and a battery of visual tests: visual acuity, contrast sensitivity, illusion of length perception, and Ishihara test. Specifically in the GET, participants were required to classify cards on the basis of two reference cards (solid black-no gradient; full range black to white gradient). PSEs of the GE show no correlation with the other visual tests, revealing a divergent validity. A significant difference between young and elderly was found: contrary to our original expectations, luminosity thresholds of GE for elderly were higher than those for young, suggesting a non-direct relationship between luminosity perception and discomfort glare.

## Introduction

When light enters the eye, not all energy reaches the retina to produce a clear cut sensation, some part is scattered (Weale, 1986b) and may determine a sometimes rather annoying phenomenon defined as *glare*. During the past years many definitions of glare have been given (CIE, 1987; Mainster and Turner, 2012), but substantially, the three definitions proposed by Vos (2003) turn out to be the most useful for categorizing different aspects of the glare experience. Disability glare is the *physiological glare* that generally impairs vision without causing heavy discomfort. On the contrary, *discomfort glare*, is a brightness phenomenon that causes annoyance: an uneasy veiling that, however, does not cause heavy visual impairment. Finally, *dazzling glare* is a different phenomenon of discomfort that emerges in a bright field of view in large retinal areas producing squinting, annoyance and aversion.

Age related changes in visual function have been reported in different domains (Pitts, 1982; Faubert, 2002). Every structure in the peripheral visual system undergoes some degree of change related to age (Spear, 1993). As an example, anatomical changes with age occur in photoreceptor rod numbers (Curcio et al., 2000), in the crystalline lens (Glasser and Campbell, 1998) and in other structures. Changes were found in several psychophysical functions: visual acuity, contrast sensitivity function, color perception and mid-level perception, such as motion, symmetry, and depth perception (Weale, 1986a; Faubert, 2002).

Along with the aforementioned age related changes, also modifications in glare sensitivity were found, with increasing sensitivity to glare in senior people (Wolf, 1960). Increased sensitivity to glare is also associated with posterior subcapsular cataracts beyond the very early stage (Lasa et al., 1992, 1993). Along with an increasing susceptibility to glare, it was found that also recovery time from dazzling and disability glare increased (Van Den Berg et al., 2009). For these reasons glare sensitivity and recovery to glare were recently included in the European criteria for driving licenses (Commission Directive 2009/113/EC, 2009).

Along with the physical and physiological studies of glare phenomena, there are perceptual studies that address a particular perceptual phenomenon of diffused brightness enhancement (Gori and Stubbs, 2006; Spillmann et al., 2010) in which one is dubbed as *glare effect* (Zavagno, 1999; Zavagno and Daneyko, 2017). In its standard form, the glare effect (GE) is a brightness illusion composed of four adjacent squares in which a white central region appears self-luminous when surrounded by linearly decreasing luminance ramps (Zavagno and Caputo, 2001). Reports talk about a self-luminous mist spreading out from the central square that is perceived as a light source. The figure is rather interesting because it gives rise to the perception of self-luminosity or glow in absence of true light emission in the case of opaque reflecting surfaces, or with low light emission in the case of computer generated displays (Todorović, 2006). It has been hypothesized that this is possible because the GE partially simulates at a distal level what happens at a proximal level when the eye is invested by light of high intensity (Zavagno and Caputo, 2005). Hence, the advantage of using this type of illusion to study dimensions such as luminosity, glare, and visual uneasiness is related to the fact that physiologically harmless luminance levels can be employed in experiments to study people’s responses to light sources in different types of tasks and in relation to different visual dimensions.

Our interests come from the fact that the GE seems to be another counterpart of glare, which is purely perceptual and not directly linked to what we may call physical discomfort glare. Based on the fact that older subjects show high discomfort glare, we hypothesize high sensitivity to GE (lower threshold) on older subjects. Based on GE, our aim was to build a practical “paper and pencil” tool that measures a perceptual process of glare in order to investigate the change of GE perception during the lifespan.

## Method

### Subjects

Participants were two groups of adults: young (30 participants, mean age = 23.3, *SD* = 2.4, range 20–29) and aged (20 participants, mean age = 64.8, *SD* = 3.9, range 60–72). All participants had no current or previous neurological or psychiatric disorders. This research was conducted in accordance with the guidelines outlined in the Declaration of Helsinki and was approved by the local ethical committee.

### Stimuli

The Glare Effect Test (GET) was constructed as a paper test to easily measure perceived luminosity thresholds for the GE in a natural setting without using artificial screens. Luminosity thresholds applied in this context are defined as when an opaque surface starts to appear as self-luminous, i.e., as glowing (Bonato and Gilchrist, 1994, 1999; Zavagno and Caputo, 2001). GET consists in 101 plastic cards of 12 cm × 12 cm in which reflectance ramps of the GE figure (10.5 cm × 10.5 cm) change gradually from solid black (card 0) to full black-to-white (card 101) in smooth steps. GE cards were created using Adobe Illustrator and printed using an Epson Stylus Photo R2400 printer on a 167 g/m^2^ Epson matt white paper. The GE does not require the employment of perfectly linear luminance ramps: any quasi-linear luminance ramps generate equally a GE (Zavagno and Caputo, 2001, 2005). Previous studies (Zavagno and Caputo, 2005; Zavagno and Bressanelli, 2008) have shown that the intensity of the GE in cross-like patterns, similar to those employed in GET, is modulated by the ratio between the range of luminance ramps and the luminance of the pattern’s background (See **Supplementary Figure S1** for some examples of GE cards).

Other than the aforementioned GET, in order to relate GE perception with other psychophysical and perceptual tasks, we applied the subsequent tests: contrast sensitivity (Pelli and Robson, 1988), visual acuity with a letter logMAR chart (Goodlite Co., Elgin, IL, United States, cod. 735000) together with an experimental crowded version with 12.5% inter optotype distance compared to the original 100%, color perception (Ishihara color tables), and an illusion of length sensitivity using Brentano-illusions (Daini et al., 2002).

### Procedure

**FIGURE 1:**
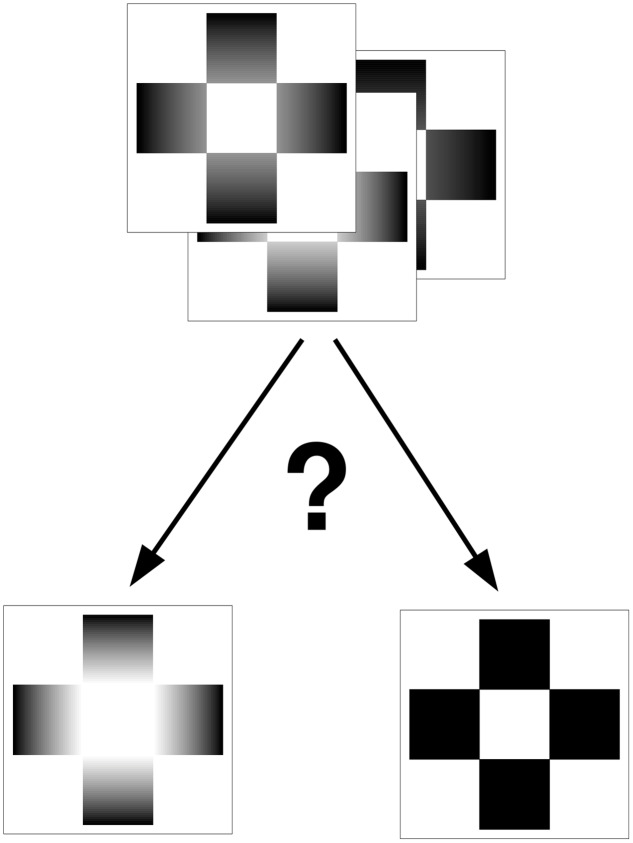
Sketch of the 2AFC method used to classify the 99 Glare cards. The participants had to indicate the similarity of luminosity of the central square compared to the two reference cards.

Participants were tested on a black table in a lab, with uniform light (61 cd/m^2^),without reflections. Referring to two reference cards (GET cards 0 and 101), participants were required to classify 99 GET cards on the basis of the brightness similarity of the central square of a card to either of the reference cards with a two-alternative forced choice (2AFC) task. The two reference cards were placed on the table at about 40 cm of distance from the eyes, and separated by 30 cm. Prior to the categorization task, participants were requested to report and describe the difference between the central squares of the two reference cards. Subsequently, the experimental task was explained using one random card from a deck of cards. Participants were instructed that they should pay attention to the central square and point to which one of the two reference cards the target appeared more similar in terms of brightness (**Figure 1**). Experimental cards were randomly presented by the examiner, one at a time and placed 10 cm above the reference cards, which were always visible. The answer for each card (99 trials) was recorded. Cards that were perceived similar to reference card 0 (solid black ramps) were assigned a score of 0, while cards similar to reference card 101 (full black to white ramp) were assigned a score of 1. These data were subsequently ordered by card number and they were fitted with a psychometric function using generalized linear model in R (R Core Team, 2017). For each subject a regression model was calculated and a specific Point of Subjective Equivalence (PSE) was extracted, along with the slope of the psychophysical function (Knoblauch and Maloney, 2012). In this way, PSEs represent a score of sensitivity to the GE, and they can be considered a threshold, which may range from 0 (no GE effect) to 99 (max GE effect); the slope of regression, instead, represents the accuracy of this estimation.

## Results

**FIGURE 2:**
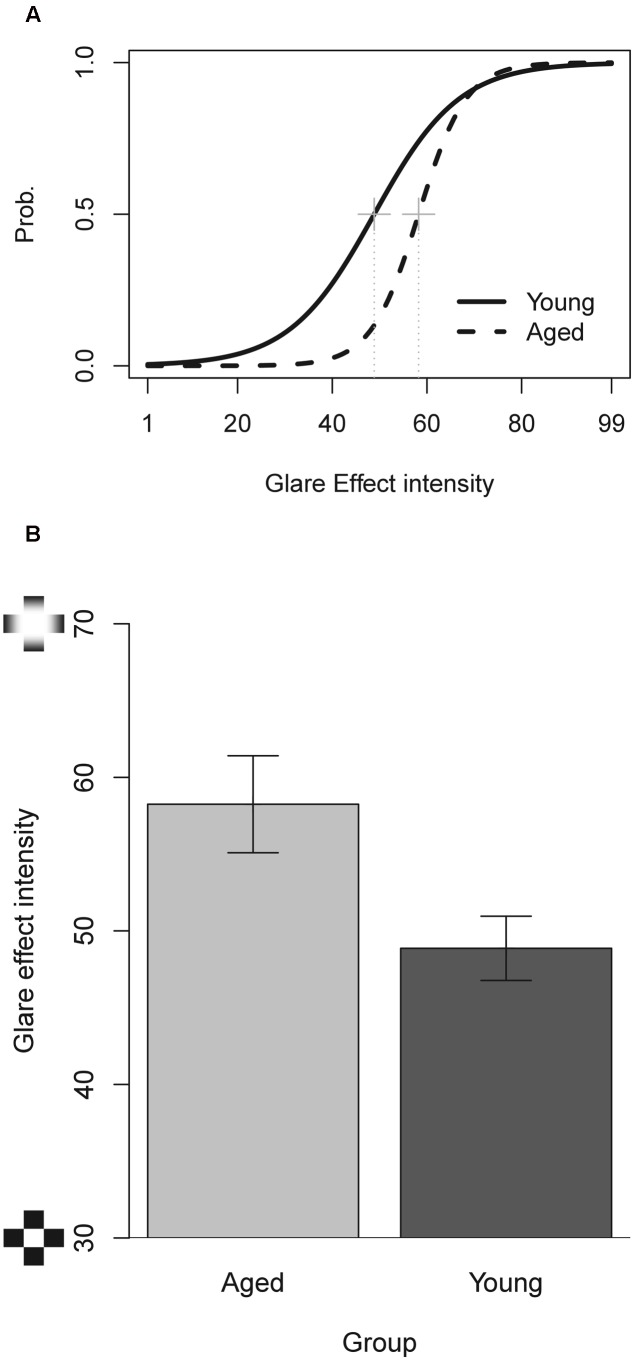
**(A)** Mean psychometric functions for the glare effect cards. **(B)** Comparison of PSE’s of the young and aged groups. Bars represent ±1 SEM.

A first analysis was carried out in order to compare PSEs of perceived luminosity thresholds. A Welch *t*-test shows a significant difference between the groups “Young” and “Aged” [*t*_(35)_ = 2.48, *p* < 0.05, -95%CI = 1.69, +95%CI = 17.07, *d* = 0.72; “Young” mean = 48.86; *SD* = 11.46; “Aged” mean = 58.24; *SD* = 14.12] showing an higher threshold for the aged group. No significant differences were found for the slopes of the regression (“Young” mean = -0.111; *SD* = 0.055; “Aged” mean = -0.199; *SD* = 0.209). The comparison of variance data between groups with Levene’s test showed a significant variances difference for Slope of regression (*p* < 0.005) (**Figure 2**).

An analysis of correlation was performed between the two GE parameters (PSE and slope) and the data for the other visual test applied. The results, even without correction for multiple comparisons, for both groups, did not show significant relationships (all *r* < 0.20 and all *p* > 0.20), revealing a divergent validity of GET.

## Discussion

The aim of this study was to build a “paper and pencil” test aimed at measuring the GE in two groups of participants that differed in age: young and aged. We found a difference between the two groups of participants, but contrary to our expectations luminosity thresholds for the “aged” group were higher than those for the “young” group, suggesting a non-direct relationship between luminosity perception and discomfort glare.

The absence of relationship between other measures suggests the divergent validity of GET. At this regard, we expected to find at least a relationship between GET thresholds and the outcome of the contrast sensitivity test (CS), because aged people present lower contrast thresholds and a higher GET threshold, two aspects potentially related. A first possible explanation to this lack of correlation is of course that the two measures are actually unrelated. A second explanation could arise from the specific measurement of contrast sensitivity: we have not measured a complete contrast sensitivity function, but only contrast in the higher spatial frequency, the correct part of the CSF spectrum that seems related to the changes during age (Derefeldt et al., 1979), but there is a possibility to find a relationship with medium but not to lower spatial frequencies (Owsley et al., 1983). In fact the complete CSF curves are considered a good psychophysical measure to evaluate disability glare (Abrahamsson and Sjöstrand, 1986). Finally, a third possible explanation could be due to the use of the Pelli Robson chart, which allows to easily detect CS deficits in a pathological population, but which might not be as accurate as we need to measure physiological CS changes in the elderly. In fact, in this view, one might hypothesize that CS deficits found with the elderly may “washout” weaker luminance ramps (low range), hence the elderly may need stronger luminance ramps (high range) to start seeing the central square of a GET card as self-luminous.

The result of a lower sensitivity (higher threshold) to glare in the aged group invalidates our initial hypothesis of a direct relationship between disability glare and the GE. This result can be interpreted in several ways. Firstly, because the GE seems to be the perceptual counterpart of glare, it is processed after receiving a “glared” image from the eye. Because older population perceived physical glare with a lower threshold, the higher perceptual threshold could be a mid–level perceptual adaptation to obtain a balanced final elaboration of the visual scene. On the other hand, high physical glare was counterbalanced by a reduced perceptual glare.

A second possible account is related to contrast sensitivity and the GET card test: limitations in contrast sensitivity by the elderly may impede the perception of lower range gradients, hence the central white targets of GET cards will be classified as similar to the full gradient card only when reflectance gradients are more pronounced. On the basis of this account, we might find that if we switch to a screen presented GET, in which actual light is emitted by the displays, thresholds for self-luminosity in the elderly would lower as contrast sensitivity is improved by the type of presentation.

Finally, the changes in GE sensitivity with age could be explained by the modulation of high cortical processes over GE perception (Leonards et al., 2005; Lu et al., 2006) and their changes with aging (Hedden and Gabrieli, 2004; Kim et al., 2016).

In perspective, based on the results obtained, more studies are needed in order to solve the different explanations given and to relate the physical and the perceptual aspects of glare.

## Author Contributions

All authors contributed to the design of the study. All authors participated in the analysis and interpretation of the data, in writing and revision of the manuscript and they approved the final version of the work.

## Conflict of Interest Statement

The authors declare that the research was conducted in the absence of any commercial or financial relationships that could be construed as a potential conflict of interest.

## Supplementary Material

FIGURE S1 Examples of GE cards with different glare effect intensity.
