# Supplementary figures and images for: The Glare Effect Test and the Impact of Age on Luminosity Thresholds
Source: Front Psychol. 2017 Jun 30;8:1132. doi: 10.3389/fpsyg.2017.01132 (PMC5492864; doi:10.3389/fpsyg.2017.01132)

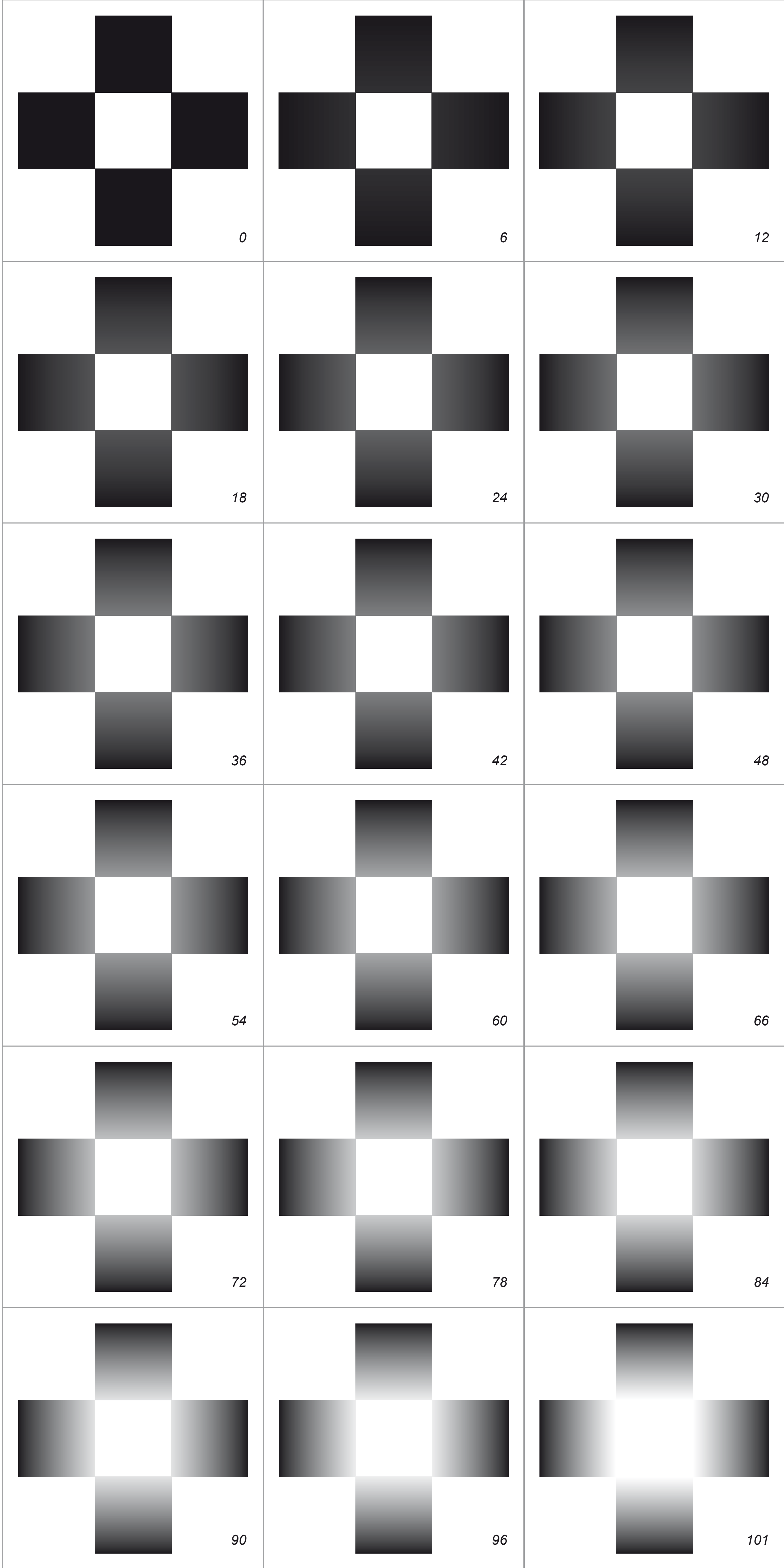

Supplement: FIGURE S1 — Examples of GE cards with different glare effect intensity. [file Image_1.PDF]
